# Dr. John Gordon Smith, on the Principles of Forensic Medicine

**Published:** 1821-09-01

**Authors:** 


*The Principles of Forensic Medicine, systematically arranged, and applied to British Practice.* By John Gordon Smith, M. D. Octavo, pp. 503. London, 1821.

As one of the great objects of legislation is the protection of human life against open violence or secret assault, so the perpetration of these acts is solemnly and severely punished, not in revenge for what is past, but as a security for the future. It is upon these melancholy occasions that medicine comes in contact with jurisprudence; though so widely separated, in general, that Blackstone considered it unnecessary for the members of the medical profession to have any knowledge of legal matters, since they are exempt from the burthen of public service, to which others are liable. As far as our *professional* character is concerned, we are perfectly of Judge Blackstone's opinion; for, in medical jurisprudence, the physician has nothing to do with the *law* of the case, but only to elucidate the *physical* circumstances, leaving all moral deductions and judicial reasonings or sentences to the constituted authorities. We cannot agree with M. Foderee, therefore, in the following sentiments:—"Beaucoup de bons praticiens," says he, "sont embarrassés quand ils sont appelés par la medicine judiciaire; *il faut connaitre les lois de son pays, les formes usitées dans les tribunaux, les termes dans lesquels un rapport doit etre conçu, suivant la disposition des lois, &c.*" Why this would be making lawyers of physicians. We really see no necessity for any *legal* knowledge in these cases. We are not examined on points of law, but on points of physiology and pathology—subjects quite comprehensive enough in themselves, Heaven knows! But then, to give this physical evidence with propriety or any thing like certainty, we must not trust to the resources of *general* professional knowledge; we must make ourselves acquainted with the relations which physiological and pathological principles bear to the facts judicially investigated—in short, we must now study Forensic Medicine, or subject ourselves to the risk of exposure in a court of justice, and consequent forfeiture of character, although perfectly qualified in our profession for all common and practical purposes, at the bed-side of sickness. This is a tax imposed upon us by the progress of civilization, the diffusion of human knowledge, and the march of science. We must submit to it, not merely with resignation, but encounter it with zeal, so as to discharge the duty with honour to ourselves and justice to the public.

But, when properly viewed, the study of forensic medicine should not be considered a hardship, nor will it be found unprofitable, even were we never to be called upon in judicial proceedings. What is it but an attentive investigation of certain physiological and pathological points which, as they do not cross our path every day in the common routine of practice, are therefore comparatively neglected, till we are accidentally roused to a sense of their importance, by some event that makes a demand on our information, for which we are not quite prepared.

The importance of this study must rise in our estimation, when we reflect that not only are our own characters at stake, but often the characters and lives of our fellow creatures. We agree with M. Fodéré that the medical practitioner should not only be possessed of medico-legal knowledge, but that he should be distinguished for probity and disinterestedness, since he occasionally holds in his hands the destiny of individuals, and the peace and honour of families, while his decisions guide the magistrate in the punishment of crime, and rescue innocence from unmerited reproach.

Our readers are all aware that, till very lately, forensic medicine was little studied in this country, and the publications on this extensive subject but few in number, and on very limited scales. The work before us is not only more comprehensive in its plan, but more complete in its execution, than any of its predecessors in the English language; and Dr. Smith deserves well of his brethren for collecting so many valuable materials, in forensic medicine, and arranging them for ready application to the exigencies of life. "In pursuing my task," says he, "I have endeavoured to gather useful information from sources where it was unexpected, as well as quarters where it had been forgotten; and many facts applied to the elucidation of these principles have been literally *found* in the chaos of recorded occurrences." We shall now proceed, without further preface, to present the reader with such notices and specimens of Dr. Smith's work, as the great variety of subjects in the volume and the narrow limits of our Journal will permit.

Our author has arranged (with a modest apology for the imperfection of the arrangement) Forensic Medicine into four distinct classes.

"I. Those which regard the extinction of human life; particularly by unusual or violent means. Such are many kinds of sudden death, and all cases of homicide.

"II. Injuries done to the person, not leading to the extinction of life. Such are disfiguring and maiming; causing diseases; the violation of females, &c.

"III. Circumstances connected with the physical system, that disqualify for the discharge of civil offices, or the exercise of social functions. Such are mental alienation, the existence of certain diseases, the want of certain organs, &c.

"IV. Whatever regards the preservation of the public health; as the proper regulations in times of public sickness, the administration of public institutions for the cure of diseases, public nuisances, and many subjects of the last importance, forming that conspicuous department, termed *Medical Police.*" 13.

Of the *last* class he does not treat at all, in the present publication; and it is to the *first* that he has paid the greatest attention, as the most important in its nature, and frequency of occurrence. The *first* class is again divided into four sections; namely, sudden death in the healthy state—death by personal agency, homicide—death by spontaneous agency, suicide—and infanticide.

I. *Sudden Death in Health.* Dr. Smith prefaces this section with many judicious observations on the phenomena of death, the certainty of this event, and those states of the living body that resemble death. It is difficult to say what *is* death, except by terming it the cessation of life—that is, a cessation of the functions of life, as respiration, circulation, sensibility—followed, after a certain time, by decomposition. But in cases of suspended animation, as in asphyxia, from hanging, drowning, inhalation of noxious gases, and some diseases, there is complete suspension of the functions characteristic of life. Here the mere examination of the body cannot be trusted to. We must enquire into the nature and duration of the causes, as *they* will greatly assist our judgment in the forensic question of death.

Dr. Smith, after making all necessary allowances for popular credulity, has no doubt but that premature interment has sometimes taken place. He states that he himself was an eye-witness of an instance on the Continent, where a poor woman "was solemnly ushered to the margin of the grave, in broad day, and whose interment would have deliberately taken place, but for the interposition of bystanders."

In the *second* chapter on sudden death, our author makes various judicious remarks on coroners' inquests, and the duties which medical men are called upon to perform on these occasions. To show the necessity of dissection in all doubtful cases, our author presents several illustrations, of which we shall here quote an example.

"A person may be carried off by apoplexy, under circumstances calculated to excite suspicion either as to the conduct of the deceased himself, or that of others, and to throw a mystery over the event, which a judicious examination may entirely remove. For instance, the usual turgidity and discolouration about the countenance may be wanting, while wounds and bruises appear in various parts of the body; and all this, upon careful examination, may admit of easy and natural explanation. A man may be overtaken with an apoplectic paroxysm in a place where there are hard or sharp objects, upon which he falls, as against furniture in a room, or among stones out of doors; he may thus receive wounds that appear extensive, and may lose a considerable quantity of blood. This would be ample cause for popular alarm and clamour, by which the scientific practitioner is fully aware that he never ought to be swayed. On dissection the real cause of death will appear, which, together with the extent and nature of the wounds, will shew that death has not been produced by external violence. A still more complicated case however may occur. It is possible that a persom in the apoplectic state may fall alive into water, and be taken out dead. In such a case we may expect that the signs of apoplexy will be manifest; and circumstantial considerations must have weight; such as the place in which the deceased was found; the appearance of the ground on the margin of the water; the previous state of his mind, health, and general circumstances; as also the degree in which the phenomena of death by submersion exist; of which mention will be made in the proper place. It is to be understood that I am talking of *Apoplexia Sanguinea.*

"Let us suppose another case, one that perhaps may never occur, but which, however extreme, is possible. A person may fall from a height in a fit of apoplexy, or in any other paroxysm that deprives him of the perception of danger and the power of avoiding it. In consequence of this, his scull is fractured, and he is found dead. It can hardly be supposed that the manner in which the fracture has been inflicted can remain a mystery; it will be seen, from the situation of the body relative to surrounding objects, that it has happened by a fall; but three questions may arise. Has the deceased accidentally come by his death in this way? Or has he sought it of his own accord? Or has he been precipitated by the agency of other persons? Between the questions of *mere* accident, and that of suicide, other persons may, perhaps, be as able to decide as ourselves; for dissection may discover nothing but the fractured scull and its consequences. Previous history must here be considered; but it may be, that on opening the cranium, evident marks of apoplexy are found, and further enquiry may lead to the conclusion that the deceased was seized with a paroxysm of this disorder in a dangerous situation, by which he received the fall in question." 39.

The author, after some pertinent remarks on the morbid appearances in apoplexy, and the various phenomena that indicate a predisposition to that disease, proceeds to lay down an outline of the medical practitioner's conduct where a person is found lying dead, and no one is forthcoming to give any information on the subject. For these instructions we refer to the work, page 40 *et seq*. What is said of the medico-legal relations of apoplexy will apply to other causes of sudden death, *mutatis mutandis*, as epilepsy, &c.

Passing over a great mass of elementary forensic matters, from page 40 to page 65, we come to an important chapter on poisoning. The deleterious substances employed for this purpose are classed by our author rather according to the kingdoms of nature whence they are procured, than according to their action on the living animal system.

> " For it is not so much with the symptoms and derangements caused by poisons, that our business lies, as with the mode of detecting their presence, or at least of ascertaining the fact of their having been administered; for which purpose, though symptoms may be useful, they are no more than auxiliaries." 66.

Still the qualities of poisons, as corrosive, astringent, acrid, narcotic, &c. are not to be overlooked, as they assist us in forming an opinion as to the fact of poison having been administered. The following extracts, as containing interesting matter in themselves, will also afford specimens of our author's manner.

> " The general symptoms that ensue when a person has taken corrosive poison, are violent pain and sense of heat in the stomach and intestines, accompanied with constriction of the mouth and fauces—frequent vomitings, often of blood, followed by bloody diarrhœa, and occasionally attended with hiccup and tenesmus. The pulse is quick, small, and hard, becoming at length imperceptible. The body becomes very cold, and suffused with cold moisture—though these symptoms vary; there being sometimes intense heat, accompanied with inextinguishable thirst. Some poisons of this class produce priapism. There is generally great anxiety and oppression at the præcordia, and fœtor of the breath. In the mean time gangrene is rapidly advancing within, although the production of this state of the parts is not required to bring on the fatal result. The countenance becomes altered and convulsed; while the internal senses remain unimpaired." 69.

The following passage forms a proper sequel to the above.

> " On examining the bodies of those who have died in this manner, the following appearances have generally presented themselves. Externally they have been found livid, with more or less of a distorted appearance about the countenance. On laying them open from the fauces downwards, the specific and immediate effect of these poisons can generally be traced. The parts over which they have passed will be found more or less excoriated, if the texture be not destroyed. In the stomach and neighbouring intestines there are generally traces of the most violent inflammation, indicated by destruction of the villous coat, and even extending to gangrenous spots and eschars—nay, frequently to absolute perforations. In various parts of the intestinal canal, constrictions also are found.

"Separation of the coats of the intestines likewise takes place; and this circumstance has been considered conclusive. But cases are on record, in which detachment of the villous coat of the stomach and intestines has taken place, without the slightest ground to suspect the administration of poison." 70.

Our author enters fully into the important subject of poisoning by arsenic, because the directions and precautions will apply, with trifling modification, to poisoning by most other mineral substances. We can only glance at a few particulars in this section of the work.

In the event of our being called to a person supposed to be poisoned, but yet alive, we should endeavour quickly to get at the remainder, if any, of the substance swallowed, and test it with such things as are nearest at hand—as lime water, or lunar caustic. If the person have vomited, we should preserve the matters ejected from the stomach for the same purpose. The symptoms and the exhibition of antidotes will strengthen our conclusions. If the individual dies, we must proceed to a more accurate investigation. The following extract will shew our author's manner of treating his subject.

"The trunk of the body is to be carefully laid open from the top of the thorax to the cavity of the pelvis, taking every precaution to wound no part of the alimentary canal. This being done, let the whole of the intestines be removed; which is to be accomplished by careful separation from their attachments; one ligature being securely placed on the upper part of the œsophagus, a second on the lower part of the intestinum rectum, and a third on the vessels that pass between the duodenum and the liver, whereby every possible precaution will be taken to guard the contents from escape. If we discover preternatural perforations in the stomach or elsewhere, ligatures (even if practicable) might be improper; we must endeavour to avoid the loss of substance through them, by attending to the position in which they are maintained during the process of dissection, and clean spunges may be applied, partly to prevent, as much as possible, the fluid from spilling, and also to absorb and preserve what portion of it does make its way through.

"While this is going on, a large earthen vessel, of a capacity sufficient to receive the viscera, should be prepared—perfectly clean and dry—to which the whole intestinal canal is to be transferred.

"Other vessels of the same kind, though not necessarily of equal dimensions, are also to be got ready. The canal being laid open throughout its whole extent, the fluid contents are to be placed in one vessel, and the solid in another. The intestines are then to be washed in distilled water, and the product of this is also to be carefully set apart. These precautionary steps being taken, we proceed to search accurately for lesions of structure, and morbid appearances; and whatever may be discovered in this way should be correctly noted down. Eschars, gangrenous inflamed and perforated spots, Orfila recommends to be removed with a portion of the parts around them, and placed in alcohol.

"The preliminary preparations for a chemical examination having been arranged, we now proceed to analyse the various substances obtained. In the first place we must search for solid particles of the arsenious acid, and if we find any, let them be tried in the various ways already described. If our search for them be unavailing, out attention must then be directed to the contents of the alimentary canal in general; and it will be a convenient rule to keep those of the stomach separate from the rest.

"Our author directs that the solid part of the contents should be boiled in ten or twelve times its weight of distilled water, for one hour, renewing the water as fast as it flies off in vapour, This liquor is to be cooled, and decanted from the residue before the tests be applied. But, as the degree of solubility of arsenious acid in water at the boiling point is stated by Orfila himself to be as one part to fifteen of water, I should think that the success of the experiment would be better insured, were the quantity of water greater than here recommended, even although the proportion of arsenic contained in the mass to be boiled, should be very small.

"The tests being applied, should they produce precipitates according to the description already given, these are to be dried, and proceeded with in the established manner of obtaining metallic arsenic.

"If the liquor, however, should offer no indications of poison in this way, we are not to rest satisfied. Orfila then directs that the mass should be *exhausted* by water, treated with caustic potass, and nitric acid gradually added, until it assumes a clear yellow colour. The excess of acid being then saturated with potass, if there be any arsenious acid present, it will combine with the potass, forming an arsenite thereof. The most delicate tests* should now be applied to the liquor; and if the precipitates give cause to suspect the presence

---

* "These are understood to be lime-water, acetate of copper, and muriate of cobalt. If chlorine be employed to remove the effect of colouring matter, the first of these will give a *white* precipitate; the second a *blue*, varying in shade according to the quantity of chlorine employed —and the last a *rose-coloured* precipitate."

of arsenious acid, let the hydro-sulphuret of ammonia and a few drops of nitric acid be applied. A yellow sulphuret of arsenic should thus be obtained, which, being first dried on a filter, then mixed with equal parts of potass and charcoal, and exposed to heat in a glass tube, will coat the sides of the tube with metallic arsenic. By breaking the glass thus coated, (if the metal cannot be well taken off) and exposing the fragments to ignition, the characteristic vapour and odour of arsenic will be developed.

"Sulphurets and hydro-sulphurets, though censured by some authors, have been recommended, and are generally esteemed as antidotes, in cases of poisoning by arsenic. Where these have been administered, the poison becomes transformed into a yellow sulphuret of arsenic. Orfila recommends the following process to be observed in such a case. Let the fluid contents of the stomach deposit all the yellow matter insoluble in water. Let this be dried on a filter, and a portion of the residue placed on burning charcoal. A smell of sulphureous acid will arise. Let another portion of the (dried) residue, finely powdered, be washed with an equal bulk of the potass of commerce, be again dried, and exposed to heat in a glass tube. Metallic arsenic will be speedily obtained by sublimation, and sulphate of potass will be formed at the bottom." 99.

The farther we have proceeded with Dr. Smith's work, the more we have become convinced that any attempt at analysis will prove abortive, since it is of that elementary and connected nature that defies any such attempt, without disfiguring the publication, or uselessly occupying space with extracts bearing no relation to each other, and consequently not calculated to prove beneficial to author or reader. Moreover, we are of opinion that, as the most complete work of the kind in the English language, no medical man, who considers it worth while to guard his professional character in a court of justice, will fail to place this Treatise on Forensic Medicine in his study, as a work of genuine merit, authentic reference, and practical application.

We subjoin a tabular view of the contents, posterior to the section on mineral poisons, the extracts introduced above being sufficient specimens of the execution of the whole.

"Sec. ii. Vegetable poisons, p. 147; § iii. Animal poisons, 178; § iv. Occult poisoning, 195.—Chapter II. Suffocation, 202: § i. Noxious inhalation, 204; § ii. Drowning, 206; § iii. Hanging, 216; § iv. Strangling, 223; § v. Smothering, 229.—Chapter III. Wounds and bruises, 233: § i. Wounds, &c. of the head, 243; § ii. Wounds, &c. of the neck, 254; § iii. Wounds, &c. of the thorax, 255; § iv. Wounds, &c. of the abdomen, 261.—Section III. Death by spontaneous agency, or Suicide, 269.—Section IV. Infanticide, 289: Chapter I. Criminal Abortion, 290; Chapter II. Infanticide, strictly so called, 308; *Survivorship*, 378.—Class II. Questions arising from injuries done to the person, not leading to the extinction of life, 286.—Sect. I. Mutilation, 388.—Sect. II. Rape, 392.—Class III. Disqualifications for the discharge of social or civil functions, 402: Section I. Mental disqualifications, 404: Mental Alienation, 405: § i. Mania, 405; § ii. Melancholia, 420; § iii. Fatuitas, 425—Sect. II. Disqualifications strictly physical, 428: Chapter I. Disqualifications for general purposes, 428: Chap. II. Disqualifications for military service, 434; Chap. III. Disqualifications for the matrimonial state, 442: § i. Impotence, 443; § ii. Sterility, 456; § iii. Diseases, 460.—Section III. Pretended disqualifications, 463: Section IV. Miscellaneous questions, 475: Chapter I. Utero-gestation, 475: § i. The phenomena of pregnancy, 476; § ii. The termination and consequence of utero-gestation, 486; § iii. Of the duration of pregnancy, 490; § iv. Supplementary observations, 493.—Chapter II. Sexual Ambiguity, 495.—Chapter III. Personal identity, 499.

